# Validation of The Umbrella Collaboration for Tertiary Evidence Synthesis in Geriatrics: Mixed Methods Study

**DOI:** 10.2196/75215

**Published:** 2025-07-08

**Authors:** Beltran Carrillo, Marta Rubinos-Cuadrado, Jazmin Parellada, Alejandra Palacios, Beltran Carrillo-Rubinos, Fernando Canillas, Juan José Baztán Cortés, Javier Gómez-Pavón

**Affiliations:** 1 The Umbrella Collaboration, C/ Ferraz, 49, Madrid, 28008, Spain, 34 637016776; 2 Department of Traumatology and Orthopedic Surgery, Hospital Central de la Cruz Roja San José y Santa Adela, Madrid, Spain; 3 Department of Geriatrics, Hospital Central de la Cruz Roja San José y Santa Adela, Madrid, Spain

**Keywords:** tertiary evidence synthesis, The Umbrella Collaboration, umbrella reviews, health research methodology, AI-assisted synthesis, evidence-based decision-making, algorithms, analytics, artificial intelligence, digital health, digital interventions, digital technology, machine learning, models

## Abstract

**Background:**

The synthesis of evidence in health care is essential for informed decision-making and policy development. This study aims to validate The Umbrella Collaboration (TU), an innovative, semiautomated tertiary evidence synthesis methodology, by comparing it with traditional umbrella reviews (TURs), which are currently the gold standard.

**Objective:**

The primary objective of this study is to evaluate whether TU, an artificial intelligence–assisted, software-driven system for tertiary evidence synthesis, can achieve effectiveness comparable to that of TURs, while offering a more timely, efficient, and comprehensive approach.

**Methods:**

This comparative study evaluated TU against TURs across 8 matched projects in geriatrics. For each selected TUR, a parallel TU project was conducted using the same research question. Outcomes of interest (OoIs), effect sizes, certainty ratings, and execution times were systematically compared. Effect sizes were assessed both quantitatively, by transforming TUR metrics to Cohen *d* and correlating them with TU’s R_TU_ metric, and qualitatively, through categorical classifications (trivial, small, moderate, and large). Certainty levels were compared by mapping Grading of Recommendations Assessment, Development, and Evaluation (GRADE) ratings and TU’s sentiment analysis scores onto a common 0‐1 scale. Execution time was measured precisely in TU, while TUR durations were estimated from literature benchmarks. Statistical analyses included chi-square tests and Spearman correlations.

**Results:**

Eight TURs in geriatrics were matched with parallel projects using TU. TU replicated 73 of the 86 (85%) OoIs identified by TURs and reported an additional 337 OoIs, representing a 4.77-fold increase in outcome identification. In the comparison of effect size classifications, full concordance was observed in 24 of the 48 (50%) cases, and consistent concordance (full plus 1-level deviation) in 45 of the 48 (94%) cases, with a moderate strength of association (Cramér *V*=0.339). The correlation of transformed certainty values between TU and GRADE yielded a statistically significant Spearman coefficient (ρ=0.446; *P*=.02). The average execution time per TU project was 4 hours and 46 minutes, compared with estimated durations of 6‐12 months for TURs.

**Conclusions:**

The TU demonstrated high concordance with TURs, replicating 73 of the 86 (85%) outcomes identified by TURs and identifying nearly 5 times as many additional outcomes. The experimental effect size metric (R_TU_) showed moderate agreement with conventional measures, and the certainty ratings derived from sentiment analysis correlated acceptably with GRADE-based assessments. While further validation is needed, TU appears to be a valid and efficient approach for tertiary evidence synthesis, offering a scalable and time-efficient alternative when rapid results are required.

## Introduction

### Background

Synthesizing evidence in health care transforms large volumes of data into actionable knowledge, enabling decisions based on the best available information. This process integrates findings from multiple sources to produce clear, accurate, and accessible summaries for clinicians, policy makers, and patients alike [1]. As a core component of knowledge translation, evidence synthesis bridges research and practice, and is essential for developing effective health policies [2,3].

Yet limited statistical and health literacy among professionals and the public often hampers the effective use of synthesized evidence [4-6]. This highlights the need for tools that democratize access to high-level information and support meaningful stakeholder participation in health care decisions [7].

In recent years, tertiary evidence synthesis, commonly known as umbrella reviews, has emerged as a third tier in the evidence hierarchy, building on primary studies and systematic reviews with or without meta-analyses (SRs/MAs). Also referred to as overviews, meta-reviews, or meta-epidemiological studies [8,9], umbrella reviews (hereafter referred to as traditional umbrella reviews [TURs]) are particularly valuable for addressing broad questions, generating rapid insights, or navigating resource constraints. Despite structured guidance from organizations such as Cochrane and the Joanna Briggs Institute [10-14], methodological inconsistency across TURs persists due to variations in implementation.

The COVID-19 pandemic underscored the need for faster synthesis methods, even at the expense of some precision [15]. In this context, The Umbrella Collaboration (TU) introduces a novel artificial intelligence (AI)–assisted approach to tertiary synthesis, combining automation and human oversight. Although tools such as Covidence, Rayyan, and DistillerSR have improved aspects of secondary synthesis [16], the application of AI to tertiary synthesis remains largely unexplored. Large language models (LLMs) such as ChatGPT show potential for automating SRs [17], but dedicated software for tertiary synthesis has yet to be established.

TU addresses this gap by offering a structured, reproducible, and fully digital alternative to TURs. It is a patent-pending software system that automates tertiary evidence synthesis through a combination of algorithmic processes, natural language processing (NLP), and selective use of LLMs. TU retrieves abstracts of SRs/MAs from MEDLINE via PubMed, using LLMs (eg, ChatGPT-4 [18]) to suggest related terms, validated by a human reviewer, to enhance search sensitivity without sacrificing specificity. Crucially, AI is limited to this initial phase; subsequent steps are fully managed by rule-based software to ensure transparency and auditability.

**Figure 1.**
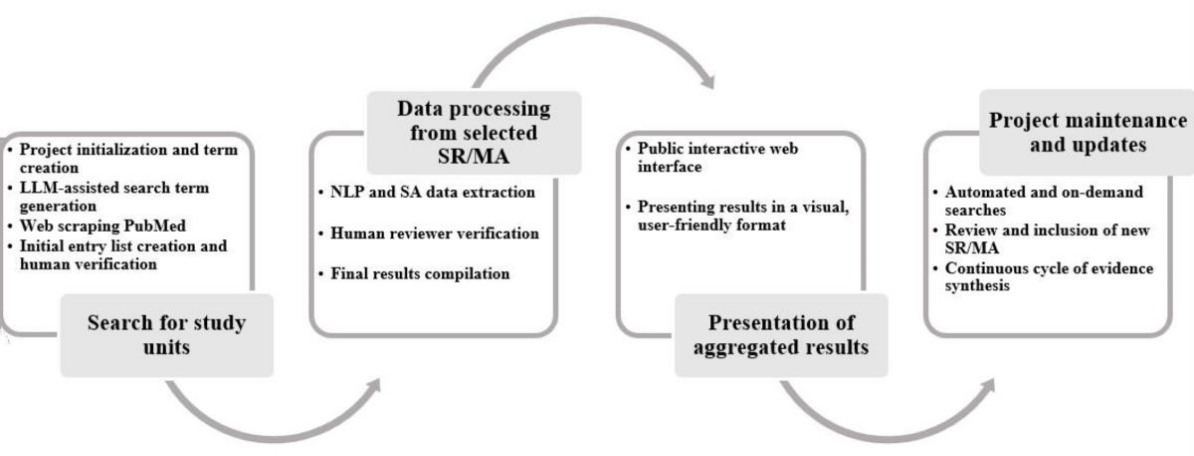
The Umbrella Collaboration workflow. LLM: large language model; MA: meta-analysis; NLP: natural language processing; SA: sentiment analysis; SR: systematic review.

TU extracts and synthesizes key information, including outcomes of interest (OoIs), effect sizes (ESs), direction, statistical significance, and a certainty estimate derived from sentiment analysis, and presents results in plain language through an interactive web platform. The system updates daily, supporting the principles of living SRs [19]. Unlike tools that assist in isolated stages of secondary synthesis, TU delivers an end-to-end solution tailored specifically for tertiary synthesis. A schematic diagram (Figure 1) illustrates the overall architecture, from automated literature retrieval to final result visualization. A more detailed technical description of the algorithms and processes is available in Multimedia Appendix 1.

As AI evolves, fully automated synthesis workflows may become feasible, but rigorous validation is essential to ensure trust, transparency, and scientific integrity.

The implementation of new methodologies in the scientific field requires a comparative validation process with established methods to confirm their reliability and effectiveness. TU, being an innovative methodology still in its theoretical-conceptual stage, must be evaluated against established methodologies. Therefore, this study aims to validate TU by comparing its performance and outcomes with the gold standard, TURs, to establish its credibility and potential superiority.

### Objectives

This study aims to evaluate whether TU, a software-driven and AI-assisted system, can produce results comparable to TURs, while offering a faster, more efficient, and potentially more comprehensive approach.

## Methods

### Study Design

**Figure 2.**
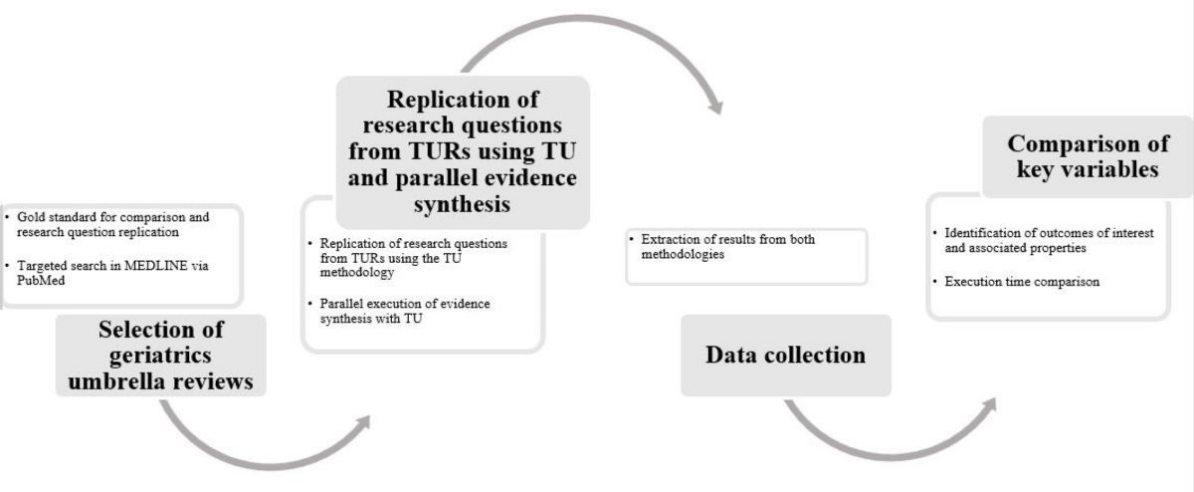
Study workflow. TU: Umbrella Collaboration; TUR: traditional umbrella review.

Figure 2 outlines the study design. Using a structured comparative approach, 8 TURs in geriatrics were selected as reference models. Each was replicated in TU using the same research questions. Key variables, including OoI, ES, certainty, and execution time, were systematically collected and compared across both methodologies.

To compare both methodologies, we conducted a targeted PubMed search to identify representative TURs in geriatrics. Rather than aiming for an exhaustive review, this focused approach was designed to select suitable benchmarks for parallel evaluation. Given the study’s aim, methodological comparison rather than comprehensive coverage, this simplified strategy was both appropriate and intentional.

### Study Variables

#### Outcomes of Interest

A key variable was the identification of OoIs, defined as specific effects evaluated by SRs/MAs addressing the same research question. We assessed concordance between TU and TURs using a concordance matrix and compared the total number of OoIs identified by each methodology through descriptive and statistical analyses.

#### Effect Size of Outcome of Interest

ES for each OoI was assessed using 2 complementary strategies: a quantitative comparison of numerical values and a qualitative classification into standard categories (trivial, small, moderate, and large). TU applies automated standardization using a proprietary metric (R_TU_), which converts various ES formats (eg, standardized mean difference [SMD], mean difference, risk ratio, odds ratio, hazard ratio) into a unified, weighted score for consistent synthesis. TUR-derived ES values were transformed into Cohen *d* for comparison. Spearman correlation was used to assess quantitative concordance, while a contingency matrix evaluated categorical agreement, distinguishing full, partial, and major discordance (Table 1). Based on the degree of agreement, each matched OoI was assigned to one of the following concordance levels.

**Table 1.** Concordance matrix for ES^a^.

TUR^b^ ES	TU^c^ ES			
	Trivial	Small	Moderate	Large
Trivial	Full concordance	Partial concordance (discrepancy of 1 category)	Discordance (discrepancy>1 category)	Discordance (discrepancy>1 category)
Small	Partial concordance (discrepancy of 1 category)	Full concordance	Partial concordance (discrepancy of 1 category)	Discordance (discrepancy>1 category)
Moderate	Discordance (discrepancy>1 category)	Partial concordance (discrepancy of 1 category)	Full concordance	Partial concordance (discrepancy of 1 category)
Large	Discordance (discrepancy>1 category)	Discordance (discrepancy>1 category)	Partial concordance (discrepancy of 1 category)	Full concordance

a ES: effect size.

b TUR: traditional umbrella review.

c TU: Umbrella Collaboration.

#### Full Concordance

Both TU and TUR classified the outcome in the same category (eg, trivial-trivial).

#### Partial Concordance

The classifications differed by only 1 level (eg, trivial-small or moderate-large). These differences were considered acceptable, as they are unlikely to lead to substantial changes in interpretation or clinical decision-making.

#### Discordance

The classifications differed by 2 or more levels (eg, trivial-moderate or small-large), representing a more substantial methodological discrepancy.

#### Consistent Concordance

A combined category including both full and partial concordance, reflecting a general alignment between methodologies even when minor categorical differences were present.

### Certainty of Evidence

TU estimates the certainty of evidence for each OoI using automated sentiment analysis (SA) applied to SR/MA abstracts. While this approach, based on NLP and a model trained on Twitter/X data, does not replicate the multidimensional Grading of Recommendations Assessment, Development, and Evaluation (GRADE) framework [20], it offers a rapid and scalable approximation. GRADE ratings from TURs were categorized as very low to high, while TU’s SA scores (ranging from −1 to +1) were normalized to a 0‐1 scale using the following formula: (SA score + 1)/2. GRADE levels were similarly mapped to enable direct comparison and statistical concordance analysis.

### Execution Time

The execution time of each methodology was assessed, with TU providing exact time measurements and TURs relying on an estimated time frame of 6-12 months based on existing literature.

### Data Collection and Research Question Replication

A targeted PubMed search using the terms “umbrella” AND “geriatric” was conducted to identify suitable TURs, which served as benchmarks. Their research questions were replicated in TU without modification. TU then applied automated searches and synthesis using NLP, web scraping, sentiment analysis, and machine learning, with human oversight for verification and extraction. Data from both methodologies were systematically collected to compare outcomes, ESs, certainty, and execution time.

TU relied solely on abstracts due to practical constraints, such as limited access to full texts and the aim to minimize language bias, as all MEDLINE abstracts are in English. Although abstracts may omit methodological details, TU uses structured extraction criteria to capture key information. This study explicitly evaluates whether abstract-based synthesis in TU can yield conclusions comparable to those from full-text TURs.

### Statistical Analysis: Data Analysis and Statistical Methods

Results from TU and TURs were compared using contingency tables for outcomes and ESs. Certainty scores from both methods were transformed to a 0‐1 scale for direct comparison. Chi-square tests assessed differences between methodologies, and Spearman correlations evaluated the association between TU certainty estimates and GRADE ratings.

### Ethical Considerations

This study did not involve human participants, personal data, or animals. All data were obtained from previously published studies and analyzed in accordance with established ethical standards for secondary data analysis. Therefore, ethical review or approval was not required. This is consistent with the institutional policy of Universidad Alfonso X El Sabio (Villanueva de la Cañada, Madrid, Spain), which exempts research based solely on publicly available, nonidentifiable data from review by the institutional review board.

## Results

### Identification of Traditional Umbrella Reviews in Geriatrics as Reference Models

**Figure 3.**
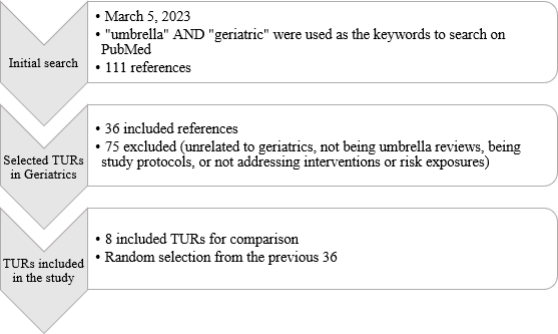
Flow diagram of the search and selection process for traditional umbrella reviews (TURs) in geriatrics.

To establish reference models, we conducted a PubMed search on March 5, 2023, using the terms “umbrella” AND “geriatric,” which yielded 111 results. After excluding 75 records for irrelevance or ineligibility, 36 TURs remained. From these, 8 were randomly selected, with a preference for recent publications, to serve as comparators for TU [21-28]. The selection process is summarized in Figure 3 and Multimedia Appendix 2.

### Identification of Outcomes of Interest

Across the 8 comparative projects, TURs identified 86 OoIs, while TU identified 410 for the same research questions. TU replicated 73 of the 86 (85%) TUR OoIs and missed 13 (15%). Conversely, only 73 out of 410 (17.8%) TU’s OoIs were reported by TURs, leaving 337 (82.2%) additional OoIs uniquely identified by TU (Table 2).

The identification gain factor, calculated as the ratio of OoIs identified by TU to those identified by TURs, quantifies TU’s broader retrieval capacity. TU consistently identified more OoIs across all projects, with an overall gain factor of 4.77 (ie, nearly 5 times more outcomes). Project-specific gain factors ranged from 1.87 to 18.63 (Table 3).

**Table 2.** Global summary table of OoIs^a^ identified by TURs^b^ and TU^c^: totals, matches, and discrepancies.

	Project								Total (TUR: 86 OoIs; TU: 140 OoIs)
	1 [22] (TUR: 5 OoIs; TU: 12 OoIs)	2 [27] (TUR: 38 OoIs; TU: 71 OoIs)	3 [23] (TUR: 7 OoIs; TU: 34 OoIs)	4 [21] (TUR: 10 OoIs; TU: 22 OoIs)	5 [28] (TUR: 4 OoIs; TU: 25 OoIs)	6 [24] (TUR: 8 OoIs; TU: 149 OoIs)	7 [25] (TUR: 6 OoIs; TU: 49 OoIs)	8 [26] (TUR: 8 OoIs; TU: 48 OoIs)	
TUR OoI identified by TU, n (% TUR)	5 (100.0)	30 (78.9)	5 (71.4)	7 (70.0)	4 (100.0)	8 (100.0)	6 (100.0)	8 (100.0)	73 (84.9)
TUR OoI not identified by TU, n (% TUR)	N/A^d^	8 (21.1)	2 (28.6)	3 (30.00)	N/A	N/A	N/A	N/A	13 (15.1)
TU OoI identified by TUR, n (% TU)	5 (41.7)	30 (42.3)	5 (14.7)	7 (31.8)	4 (16.0)	8 (5.4)	6 (12.2)	8 (16.7)	73 (17.8)
TU OoI not identified by TUR, n (% TU)	7 (58.3)	41 (57.7)	29 (85.3)	15 (68.2)	21 (84.0)	141 (94.6)	43 (87.8)	40 (83.3)	337 (82.2)

a OoI: outcome of interest.

b TUR: traditional umbrella review.

c TU: Umbrella Collaboration.

d N/A: not applicable.

**Table 3.** OoIs^a^ identified by TU^b^ and TURs^c^, additional OoIs identified by TU, and identification gain factor.^d^

Project	Number of OoIs identified by TU, n (n=410)	Number of OoIs identified by TUR, n (n=86)	Additional OoIs identified by TU, n (n=324)	Identification gain factor of OoIs by TU compared with TUR
Project 1: Veronese et al (2023) [22]	12	5	7	2.40
Project 2: Marx et al (2021) [27]	71	38	33	1.87
Project 3: Shen et al (2022) [23]	34	7	27	4.86
Project 4: Conneely et al (2022) [21]	22	10	12	2.20
Project 5: Gazzaniga et al (2023) [28]	25	4	21	6.25
Project 6: Musazadeh et al (2023) [24]	149	8	141	18.63
Project 7: Veronese et al (2021) (1)	49	6	43	8.17
Project 8: Veronese et al (2021) (2)	48	8	40	6.00

a OoI: outcome of interest.

b TU: Umbrella Collaboration.

c TUR: traditional umbrella review.

d The total identification gain factor of OoIs by TU compared with TUR is 4.77.

### Concordance in Effect Size Classification of Outcomes of Interest

To assess agreement, ESs for 48 matched OoIs were compared between TURs and TU. TUR-reported ESs were standardized (Cohen *d*) for comparison with TU’s R_TU_ metric. TURs classified most OoIs as small (n=23, 48%) or trivial (n=16, 33%). TU showed a similar pattern, with 26 (54%) classified as small and 17 (35%) classified as trivial. Moderate effects were less frequent—TUR: n=7 (15%); TU: n=5 (10%)—and large effects were rare, with TUR reporting 2 (4%) and TU reporting none.

Table 4 summarizes the categorical agreement in ES classification. TU labeled 17 out of 48 (35%) OoIs as trivial; of these, 10 out of 17 (59%) matched TUR classifications, while 7 out of 17 (41%) were classified as small. Among OoIs, TU classified as small (26/48, 54%), half-matched TURs, with the rest spread across trivial (5/26, 19%), moderate (6/23, 23%), and large (2/26, 8%) classifications. For the 5 of 48 (10%) OoIs labeled as moderate by TU, TURs agreed in only 1 case, classifying most as small or trivial.

Of the 48 OoIs analyzed, full concordance in ES classification was found in 24 (50%) cases and partial concordance (1-level difference) in 21 (44%) cases. Only 3 (6%) cases showed major discordance. Overall, consistent classification (full + partial) was observed in 45 out of 48 (94%) samples (Table 5).

**Table 4.** Contingency table of effect size classification: TUR^a^ versus TU^b^ (qualitative categories).^c^^,^^d^

TUR effect size	TU effect size, n (%)				Total, n (%)
	Trivial	Small	Moderate	Large	
Trivial	10 (59)^e^	5 (19)^f^	1 (20)^g^	N/A^h^	16 (33)
Small	7 (41)^f^	13 (50)^e^	3 (60)^f^	N/A	23 (48)
Moderate	N/A	6 (23)^f^	1 (20)^e^	N/A	7 (15)
Large	N/A	2 (8)^g^	N/A	N/A	2 (4)
Total	17 (35)	26 (54)	5 (10)	N/A	48 (100)

a TUR: traditional umbrella review.

b TU: Umbrella Collaboration.

c Cramér *V* was the effect size measure.

d Pearson *χ*2=11.03^i^ (*P*=.09), *V*=0.339 (moderate).

e Full concordance.

f Partial concordance (discrepancy of 1 category).

g Discordance (discrepancy of more than 1 category).

h N/A: not applicable.

i Marginally significant (*P*<.10).

**Table 5.** Concordance levels in effect size classification: frequency and cumulative distribution.

Classification	Absolute frequency, n	Cumulative absolute frequency, n	Relative frequency, n/N (%)	Cumulative relative frequency, n/N (%)
Different effect size	24	24	24/48 (50)	24/48 (50)
Same effect size	24	48	24/48 (50)	48/48 (100)
Same effect size	24	24	24/48 (50)	24/48 (50)
One-level discrepancy	21	45	21/48 (44)	44/48 (94)
Discrepancy>1 level	3	48	3/48 (6)	48/48 (100)

### Correlation of Quantitative Effect Sizes Between TU and TURs

**Figure 4.**
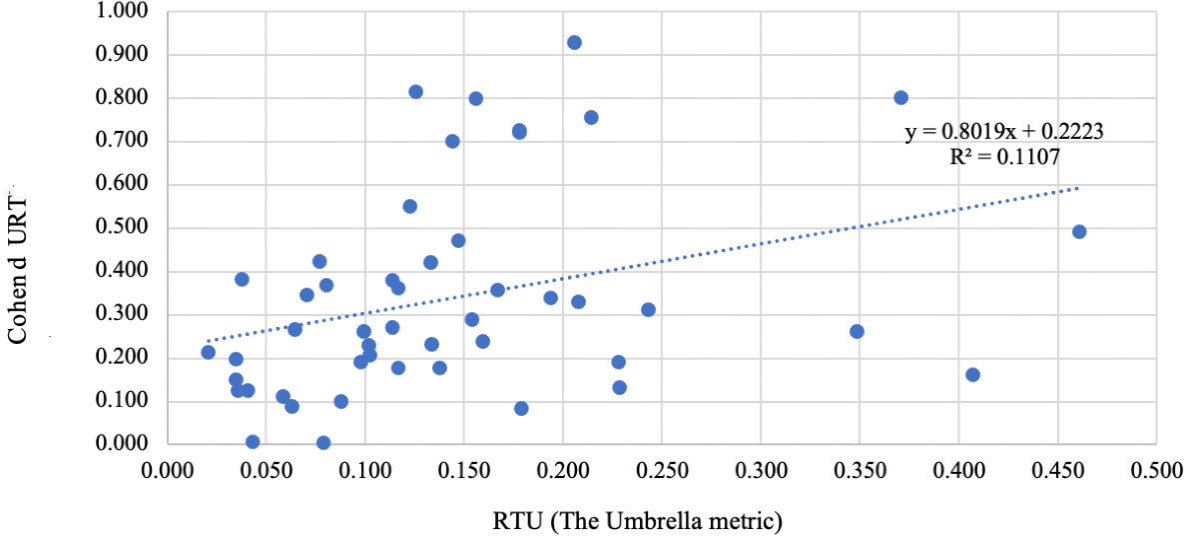
Correlation of quantitative effect sizes between Umbrella Collaboration and traditional umbrella reviews.

A Spearman correlation assessed the relationship between TU and TUR ESs (R_TU_ vs Cohen *d*), yielding ρ=0.399 (*P*=.005), indicating a statistically significant, low-to-moderate positive correlation. The nonparametric approach was chosen to ensure robustness against outliers and deviations from normality, as both the TU and TUR ES distributions significantly deviated from normality according to the Shapiro-Wilk test (*W*=0.874 and 0.847, respectively; *P*<.001 for both cases). The nonparametric approach ensures robustness against outliers and nonnormality. A scatter plot with a regression line (Figure 4) confirmed this trend, although the explained variance was modest (*R*^²^=0.111).

### Concordance in Transformed Certainty Scores Between TU and TURs

**Figure 5.**
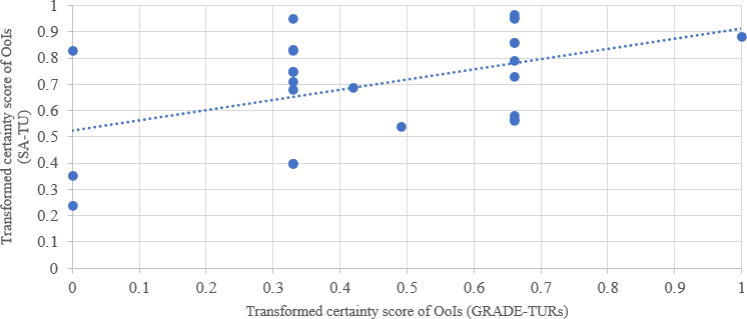
Scatter plot of transformed certainty scores for outcomes of interest (OoIs; n=25) assessed by traditional umbrella reviews (TURs; Grading of Recommendations Assessment, Development, and Evaluation [GRADE]) and Umbrella Collaboration (TU; sentiment analysis [SA]).

We compared transformed certainty scores between TU and TURs using GRADE in 5 of the 8 included reviews, yielding 25 matched OoIs. Figure 5 illustrates the relationship between GRADE-based scores (x-axis) and TU’s sentiment-based estimates (y-axis), both normalized to a 0‐1 scale for direct comparison.

A Spearman correlation assessed the relationship between transformed certainty scores from TURs (GRADE) and TU (sentiment analysis). Due to nonnormal distributions of the transformed certainty scores, Shapiro-Wilk *W*=0.874 (*P*=.005) for TUR and *W*=0.928 (*P*=.049) for TU, a nonparametric test was used. A Spearman correlation between the transformed certainty scores assigned by TURs and TU yielded ρ=0.446 (*P*=.02), indicating a statistically significant, moderate positive association that supports concordance between both approaches.

### Execution Time of TU Projects

Execution time was assessed only for TU, as none of the selected TURs reported this metric. While TURs typically take 6‐12 months, TU projects were completed in a mean time of 4 hours and 46 minutes (SD 2 hours and 30 minutes), with a median of 4 hours and 36 minutes. Completion times ranged from 1 hour and 34 minutes [28] to 10 hours and 8 minutes [24] (Table 6).

**Table 6.** Project execution time, search parameters, and reference overlap between TU^a^ and TUR^b^ methodologies.

Project	Search date	TU execution time (h:min:s)	Number of TU search terms, n	Number of references in TUR, n	Number of references in TU, n	Reference overlap, n/N (%)
Project 1: Veronese et al [22]	August 1, 2023	2:37:27	16	5	8	5/5 (100)
Project 2: Marx et al [27]	May 1, 2023	4:54:57	60	15	54	15/15 (100)
Project 3: Shen et al [23]	May 1, 2023	5:29:22	19	6	18	6/6 (100)
Project 4: Conneely et al [21]	May 1, 2023	5:37:14	6	16	23	16/16 (100)
Project 5: Gazzaniga et al [28]	May 1, 2023	1:34:37	15	11	19	10/11 (91)
Project 6: Musazadeh et al [24]	November 12, 2023	10:08:24	41	37	90	36/37(97)
Project 7: Veronese et al [25]	December 12, 2023	4:17:43	53	7	35	7/7 (100)
Project 8: Veronese et al [26]	December 12, 2023	3:33:54	16	8	22	8/9 (89)

a TU: Umbrella Collaboration.

b TUR: traditional umbrella review.

## Discussion

### Principal Findings

The findings of this study support the validity of the TU as a robust and efficient methodology for tertiary evidence synthesis. TU demonstrated a high level of concordance with TURs in identifying OoIs, successfully replicating the majority of those reported in TURs while also identifying a substantial number of additional outcomes. The experimental ES metric employed by TU (R_TU_) showed a statistically significant correlation with standardized measures such as Cohen *d* and SMD, reinforcing its methodological soundness for estimating the magnitude of interventions or exposures. In addition, TU’s automated, sentiment-based certainty assessment exhibited an acceptable level of agreement with GRADE, the most widely adopted system for evaluating certainty in evidence synthesis. One of the most notable advantages of TU lies in its execution time: while TURs typically require several months to complete, TU was able to generate comprehensive results within hours. This efficiency, combined with TU’s demonstrated consistency and methodological coherence, highlights its potential value in settings where rapid evidence synthesis is essential or where resources are limited.

The development of TU aligns with the growing interest in AI-assisted tools for evidence synthesis, an emerging field likely to shape the future of scientific research. Rather than replacing TURs, TU is intended to complement them by offering a methodologically sound alternative in contexts where conventional approaches may be impractical or untimely. As a semiautomated platform, TU leverages NLP, sentiment analysis, and machine learning to streamline tertiary synthesis while maintaining methodological rigor. Despite its potential, TU represents a paradigm shift that may require time and educational efforts for widespread understanding and adoption. During the initial dissemination of this project, a general unfamiliarity with automated tertiary synthesis was observed, underscoring the need for clearer conceptual frameworks and greater engagement with the research community.

The identification of OoIs represents a central axis in the comparison between TU and TURs. In the context of tertiary evidence synthesis, an OoI reflects a specific finding previously evaluated in 1 or more SRs/MAs and integrated to answer a defined research question. The validity of a synthesis model depends not only on aggregating relevant data but also on faithfully capturing the main conclusions emerging from the primary literature.

Given the limited literature on tertiary synthesis, this analysis draws from well-established principles in secondary synthesis, where many of the same strengths and limitations apply. These include challenges in outcome selection, variability in inclusion criteria, and the balance between breadth and precision.

The comparative analysis revealed substantial differences in the number and breadth of OoIs identified. Across the 8 matched projects, TURs identified a total of 86 OoIs, whereas TU detected 410, yielding an identification gain factor of 4.77. TU replicated 73 out of 86 (85%) OoIs reported by TURs and contributed 337 additional OoIs. This difference does not appear to stem solely from broader search strategies or inclusion criteria. According to the Cochrane Handbook [29], umbrella reviews can pursue either broad or narrow questions [30]. While both methodologies support both approaches, several TURs in this study applied restrictive filters. For example, project 6 [24] limited both its search strategy and inclusion criteria, while projects 3 [23] and 4 [21] also applied inclusion restrictions. Nevertheless, even when only unrestricted TURs are considered, TU maintained a gain factor of 4.93, suggesting an intrinsic property of TU in capturing a greater volume of outcomes.

One explanation lies in TU’s automated and systematic extraction process, which minimizes human bias and ensures that any OoI mentioned in an abstract is captured, regardless of its perceived importance. By contrast, traditional reviews often apply subjective judgment in prioritizing outcomes, potentially omitting secondary findings. Moreover, TU is not constrained by human workload, allowing it to process larger volumes of data and extract multiple OoIs per review without sacrificing comprehensiveness.

A second consideration is the differing approach to quality assessment. TURs often prioritize high-quality SRs/MAs, limiting scope but potentially increasing rigor. TU, by relying on abstracts, does not perform formal quality appraisals. While this design choice increases the number of included studies, it should not be interpreted as a methodological flaw. Notably, up to 30% of SRs/MAs fail to report study quality assessments, and only 12.9% set quality thresholds for meta-analysis inclusion [31,32]. Furthermore, tools such as PRISMA (Preferred Reporting Items for Systematic Reviews and Meta-Analyses)-Abstract could be integrated into future versions of TU to address this limitation, though prior research has shown that abstracts often score low on PRISMA quality indicators [33-36]. Nonetheless, TU consistently identifies more OoIs based solely on abstracts than TURs do using full-text reviews.

Another factor is the role of cognitive bias in outcome selection. Human reviewers may unconsciously favor familiar or hypothesis-confirming outcomes, limiting variability. While TU still involves human oversight in the final output selection, its core processes are software-driven, applying uniform parameters across all included studies and thereby reducing cognitive distortions. For instance, whereas TURs tended to report outcomes only in 1 direction, TU captured both positive and negative effects, aligning with best-practice guidelines from the Joanna Briggs Institute and the Cochrane Collaboration, which emphasize the importance of presenting balanced results for informed decision-making [29,37].

Finally, while it is possible that TU identifies outcomes of limited clinical relevance, this risk appears minimal. All OoIs extracted by TU are derived from published SRs/MAs indexed in MEDLINE, which are assumed to meet minimum standards of relevance and methodological quality. According to the Cochrane Handbook [30], reviews should focus on critical and important outcomes, excluding trivial ones.

While a higher number of outcomes may raise concerns about information overload, it is important to consider the primary purpose of tertiary synthesis: to provide a comprehensive and structured overview of the available evidence landscape. TU does not aim to guide individual clinical decisions but rather to inform stakeholders of the full scope of existing research. In this context, the inclusion of a larger set of outcomes enhances transparency and facilitates a more complete understanding of the literature. A formal qualitative appraisal of these additional outcomes, although beyond the scope of this study, represents a valuable direction for future research.

The study examined the concordance and correlation of ES metrics for OoIs between TU and TURs, using a unified set of outcomes assessed by both methodologies. TU incorporates a novel metric, R_TU_, specifically developed to standardize and synthesize ESs from SRs/MAs within a tertiary synthesis framework. Unlike conventional metrics such as Cohen *d* or the SMD, R_TU_ was designed to allow direct aggregation of heterogeneous ES measures and requires empirical validation. This analysis represents the first formal evaluation of R_TU_ against traditional metrics.

In the qualitative comparison of ES categories (trivial, small, moderate, and large), TU demonstrated a high level of agreement with TURs. Full concordance, defined as an exact categorical match between the 2 methods, was observed in 24 out of 48 (50%) cases, while consistent concordance (full plus 1-level deviation) was reached in 45 out of 48 (94%) cases. Only 3 out of 48 (6%) cases showed discordance, meaning the categorical classifications differed by more than 1 level. These findings suggest that TU approximates the classification logic of TURs with a high degree of reliability, reinforcing the potential of R_TU_ to align with established interpretive frameworks.

The strength of the association between ES categorizations assigned by each methodology was evaluated using Cramér *V*, which yielded a value of 0.339. This indicates a moderate association and supports the notion that TU and TURs tend to classify the strength of associations in a comparable manner. Although the chi-square test did not reach statistical significance, likely due to the limited sample size and sparse contingency cells, Cramér *V* remains a valid ES measure and is less sensitive to these limitations [38]. In applied research, the strength of association can often be more informative than statistical significance, particularly when evaluating the practical utility of an experimental method [39]. It is worth noting that the statistical power of the chi-square test may have been reduced by the small number of observations and the presence of empty cells in the contingency table, both of which are known to limit test sensitivity. In line with recent critiques of overreliance on *P* values in biomedical research [40-43], the observed concordance rates and ES association offer more meaningful insights into the comparability of methods than statistical significance alone.

The quantitative comparison of ES values further supports the validity of TU. The Spearman correlation coefficient between TU and TUR values was positive and statistically significant, indicating a consistent directional relationship. Additionally, the slope of the regression line approached 1.0 (*β*=.8), suggesting that the R_TU_ metric yields ES of similar magnitude to those reported in TURs using standard metrics. This represents a marked improvement in proportionality compared with earlier regression slopes reported in other validation contexts.

However, the coefficient of determination (*R*^2^) remained modest (*R*^2^=0.11), indicating that a substantial portion of the variance in TUR ES is not explained by TU estimates. This implies that, while TU captures general trends in effect magnitude, additional factors, such as differences in underlying metric types, weighting procedures, or reviewer judgments, may influence the exact values. It also highlights the limitations of linear models in fully explaining the relationship between these 2 methods.

These findings provide preliminary but encouraging evidence for the validity of R_TU_ as a useful metric for tertiary synthesis. Despite its experimental nature, R_TU_ appears to closely approximate established metrics such as Cohen *d* and the SMD in both categorical classification and magnitude. Future studies will be needed to further evaluate the sensitivity, specificity, and contextual performance of R_TU_ across diverse synthesis domains. Nonetheless, this initial validation suggests that TU can offer a consistent and efficient alternative for evaluating and categorizing ES in large-scale evidence synthesis.

While the observed correlation between TU and TUR ESs was moderate (ρ=0.399), this result is consistent with the structural differences between the R_TU_ metric and conventional measures such as Cohen *d* and SMD. R_TU_ is a proprietary, automated metric specifically developed for abstract-level synthesis, prioritizing scalability and consistency over statistical precision at the study level. It does not incorporate weighting by sample size or variance and relies on normalized effect descriptors extracted from secondary sources. These foundational differences partly account for the limited shared variance (*R*^2^=0.11). However, in the context of tertiary synthesis, where the goal is to identify trends and prioritize outcomes across a broad evidence base, R_TU_ provides an acceptable and interpretable proxy. Moreover, this divergence has limited implications for clinical decision-making, as TU is designed to support synthesis and orientation at the field level, rather than to inform individual patient decisions. Future work may explore hybrid models that integrate additional contextual parameters to enhance concordance while preserving automation.

In this study, certainty assessments were compared exclusively among those TURs that applied the GRADE framework, a well-established and widely endorsed method for evaluating the certainty of evidence [44]. Although other appraisal tools exist, such as the statistical grading criteria proposed by Papatheodorou [45] or the methodological assessment framework AMSTAR 2 [46], these differ conceptually from the certainty model implemented in TU. Papatheodorou’s system, despite its structure, has been criticized for relying on arbitrary statistical thresholds and for lacking sensitivity to clinical relevance and risk of bias [47]. AMSTAR 2, in turn, focuses on the methodological quality of the SRs/MAs themselves, rather than the certainty of individual outcomes. By contrast, TU’s approach aligns more closely with GRADE principles by estimating certainty at the outcome level, albeit through an innovative method based on an automated sentiment analysis of SR/MA abstracts. Originally, the GRADE system referred to “quality of evidence,” which denoted the degree of confidence in the validity of study findings to inform clinical decisions. Over time, this evolved to “confidence in estimates,” and more recently, to “certainty of evidence,” a term now widely accepted as it better reflects both the trust in effect estimates and their applicability in practice [20,48].

The comparison between transformed certainty scores, standardized to a 0‐1 scale, for outcomes evaluated by TU and those rated using the GRADE framework provides important insights into the potential validity of TU’s automated approach. The observed positive and statistically significant correlation suggests that TU is capable of approximating GRADE-based judgments, thereby reinforcing the conceptual alignment between the 2 methodologies. However, this result must be interpreted with caution. The number of matched outcomes was limited, which restricts generalizability and underscores the need for further validation in broader domains and larger datasets. Crucially, we recognize that TU’s sentiment-based certainty estimation cannot replicate the multidimensional rigor of GRADE, which accounts for factors such as study limitations, inconsistency, indirectness, imprecision, and publication bias. The use of a general sentiment analysis model, originally trained on nonmedical texts, represents a methodological limitation. In this context, TU’s certainty scoring should be viewed as a preliminary proxy, not a substitute for comprehensive manual assessments. Nevertheless, the approach offers a scalable and fully automated alternative capable of supporting rapid synthesis at low resource costs. Despite its current limitations, TU provides a complementary framework that aligns with the growing need for efficient, transparent, and reproducible tools in evidence synthesis. The correlation observed with GRADE invites further research into the integration of automated text-based analyses with established appraisal frameworks. As these technologies evolve, ongoing refinement and external validation will be critical to define their appropriate role in supporting scientific practice.

Execution time is a critical but often overlooked dimension in the evaluation of evidence synthesis methodologies. TURs rarely document their execution timelines, a pattern that was consistent among the TURs included in this study. By contrast, the TU methodology records execution time precisely, from the initiation of the first search term to the completion of the first version of the synthesis project, excluding any subsequent updates in this study. This level of detail in time tracking is unprecedented in both secondary and tertiary syntheses and represents a methodological innovation that may become increasingly relevant with the integration of automation tools. As a result, traditional timelines measured in months or years may soon be replaced by more accurate, time-stamped process metrics.

Given the lack of concrete time data from TURs, direct comparisons were not feasible. However, the execution times recorded for TU are sufficiently notable to highlight its efficiency. TU projects were completed within a range of 1.5-10 hours, substantially shorter than the typical duration for TURs, which is estimated to range between 6 and 12 months. This is also well below the average time frames reported for SRs/MAs, which commonly take 1-2 years [49], or for accelerated alternatives such as rapid reviews, which range from 5 to 12 weeks [50], and even the Two-Week SR approach, which targets completion in 11 working days [51]. The remarkable speed of TU is a key strength, particularly in light of the growing international demand for agile evidence synthesis methods. Organizations such as the WHO [52], Cochrane Collaboration [53], and Joanna Briggs Institute [15] have emphasized the need for rapid yet valid synthesis approaches, an objective that TU appears to fulfill effectively in the context of tertiary reviews.

It is important to note that the time measured in this study refers solely to the initial execution phase of TU projects and does not account for later updates. However, TU includes a novel updating system that keeps projects active beyond their initial completion. The platform automatically checks daily for new SRs/MAs that match the predefined search criteria. When new studies are identified, a human reviewer is notified and can assess their eligibility. If approved, the system integrates the new results into the existing synthesis following revision and confirmation. While no equivalent system currently exists in traditional tertiary synthesis, the need for regular updates is well documented in evidence-based practice. In SRs/MAs, updates are typically recommended every 2 years or in response to emerging evidence, yet compliance with this standard is often poor [54-57]. TU’s automated updating mechanism represents a major advantage in maintaining the relevance and accuracy of results, particularly in fast-evolving fields.

Notably, projects with a higher number of included references required longer execution times, which is expected given the additional workload involved in processing more SRs/MAs. Although TU significantly streamlines the synthesis process, the complexity and volume of information remain factors that influence total execution time, particularly when human oversight is still involved. Looking ahead, TU is being developed to support full automation of SR/MA data analysis, eliminating the need for human intervention. This transition will depend on training algorithms through semiautomated projects, progressively improving accuracy via machine learning.

Interestingly, the number of search terms used did not appear to correlate with execution time (unpublished data), suggesting that TU’s algorithms can manage variable search complexity efficiently. In traditional methodologies, the search phase is often complex and conducted by specialized information professionals using advanced Boolean strategies across multiple databases [58-60]. By contrast, TU simplifies this process by using basic search terms exclusively in PubMed, automatically generated and refined through generative AI. This shift toward simplified, automated search strategies opens new avenues for exploring the adequacy and efficiency of noncomplex methods in evidence synthesis. It also raises important questions about the continued necessity of highly complex search protocols, suggesting that AI-assisted models may offer comparable effectiveness in certain contexts [61-63].

Ultimately, the short execution times observed with TU not only improve the efficiency of tertiary synthesis but also enhance the feasibility of rapidly delivering high-level evidence. In settings where timeliness is essential, this temporal advantage may outweigh minor trade-offs in other methodological dimensions. This capability holds particular promise for public health and health care decision-making, where fast access to up-to-date, high-quality evidence is crucial. TU, therefore, emerges as a promising tool in scenarios where speed is a priority, offering a pragmatic balance between rapid synthesis and analytical depth, while still requiring further validation against established gold standards.

### Limitations

One key limitation is the small number of TURs included—only 8, randomly selected from 36 eligible reviews. Although intended to be representative, the lack of formal randomization introduces potential selection bias. The limited sample may not reflect the full methodological or topical diversity of umbrella reviews in geriatrics. However, all selected TURs were recent (2021‐2023), which enhances comparability by aligning with current evidence synthesis standards. Another limitation is the lack of blinding: the lead investigator selected and analyzed the TURs, potentially introducing bias. Prior knowledge of TUR findings could have influenced TU outputs. However, the heterogeneity of outcomes across TURs reduces this risk, and TU’s algorithm-driven, semiautomated workflow limits subjective influence. Results were largely determined by predefined rules, adding neutrality to the process.

The decision to utilize only 1 database, MEDLINE via PubMed, in TU is both a recognized limitation and a deliberate choice shaped by resource constraints and technical considerations. Although SRs typically require searching multiple databases to capture all relevant literature [64], our approach focuses on testing whether TU can achieve outcomes comparable to TURs despite this limitation. A pilot study provided preliminary evidence of PubMed’s strong coverage, but further validation through TU is necessary to confirm its applicability across different domains. Although searching multiple databases is often recommended to reduce language and indexing biases, particularly regarding non-English literature [65,66], TU mitigates some of these biases by focusing on abstracts in English, as all PubMed abstracts are provided in English regardless of the original publication’s language. Nonetheless, the absence of Chinese databases in our approach remains a notable limitation, given that only a small proportion of Chinese journals are indexed in MEDLINE.

Relying solely on abstracts in TU was a deliberate choice to test whether tertiary synthesis can be performed efficiently, with minimal resources, and without requiring advanced methodological expertise. While abstracts may omit key details (eg, certainty ratings, ES calculations, risk of bias), TU’s structured, automated approach aims to generate clinically useful insights under standardized conditions. This approach offers 2 main advantages: (1) reduced language bias because all MEDLINE abstracts are in English; and (2) greater feasibility, as full-text access often requires costly subscriptions.

This pilot study focused on geriatrics to ensure methodological clarity and feasibility during initial validation. Limiting the scope allowed for controlled comparisons between TU and TURs. Future research should expand TU’s application to other medical fields (eg, cardiology, psychiatry) and to nonmedical domains such as education or sociology, enabling stepwise validation before broader implementation.

### Conclusions

This study provides preliminary evidence supporting the validity of the TU as a complementary, semiautomated tool for tertiary evidence synthesis. In a set of comparative projects, TU demonstrated a high level of concordance with TURs in identifying OoIs, estimating ESs, and assessing certainty, while offering substantially shorter execution times.

Although the methodology remains experimental and further validation is required across broader contexts, the results suggest that semiautomated approaches such as TU may represent a promising step toward more efficient, scalable, and continuously updatable models of evidence synthesis. TU does not aim to replace traditional methods but to provide a practical alternative in settings where time, resources, or responsiveness are critical.

Future studies with larger datasets, enhanced blinding procedures, and expanded topic areas will be essential to confirm these findings and further explore the potential role of TU in the evolving landscape of evidence-based research.

## Supplementary material

10.2196/75215 Multimedia Appendix 1 Practical overview of The Umbrella Collaboration platform.

10.2196/75215 Multimedia Appendix 2 Traditional umbrella reviews (TURs) identified in the initial search and TURs selected for comparative analysis.

## Abbreviations

AI: artificial intelligence
ES: effect size
GRADE: Grading of Recommendations Assessment, Development, and Evaluation
LLM: large language model
MA: meta-analysis
NLP: natural language processing
OoI: outcome of interest
PRISMA: Preferred Reporting Items for Systematic Reviews and Meta-Analyses
SMD: standardized mean difference
SR: systematic review
TU: Umbrella Collaboration
TUR: traditional umbrella review

## References

[1] (2018). Evidence synthesis for policy: a statement of principles - The Royal Society 2018. International Network for Governmental Science Advice.

[2] World Health Organization (WHO) (2005). World health organization knowledge management strategy. WHO.

[3] Cottrell E, Whitlock E, Kato E (2014). Defining the Benefits of Stakeholder Engagement in Systematic Reviews.

[4] Sørensen K, Van den Broucke S, Fullam J (2012). Health literacy and public health: a systematic review and integration of definitions and models. BMC Public Health.

[5] Martínez-García JJ, Canizalez-Román A, Velázquez-Román JA, Flores-Villaseñor HM, León-Sicairos NM (2021). Evaluación del conocimiento de métodos básicos de epidemiología e investigación en médicos residentes. Rev Médica Univ Autónoma Sinaloa REVMEDUAS.

[6] Baccolini V, Rosso A, Di Paolo C (2021). What is the prevalence of low health literacy in European Union member states? A systematic review and meta-analysis. J Gen Intern Med.

[7] Baumann LA, Reinhold AK, Brütt AL (2022). Public and patient involvement in health policy decision-making on the health system level - a scoping review. Health Policy.

[8] Biondi-Zoccai G (2016). Umbrella Reviews: Evidence Synthesis with Overviews of Reviews and Meta-Epidemiologic Studies.

[9] Choi GJ, Kang H (2023). Introduction to umbrella reviews as a useful evidence-based practice. J Lipid Atheroscler.

[10] Fusar-Poli P, Radua J (2018). Ten simple rules for conducting umbrella reviews. Evid Based Mental Health.

[11] Aromataris E, Fernandez R, Godfrey CM, Holly C, Khalil H, Tungpunkom P (2015). Summarizing systematic reviews: methodological development, conduct and reporting of an umbrella review approach. JBI Evid Implement.

[12] Cant R, Ryan C, Kelly MA (2022). A nine‐step pathway to conduct an umbrella review of literature. Nurse Author Ed.

[13] Belbasis L, Brooker RD, Zavalis E, Pezzullo AM, Axfors C, Ioannidis JP (2023). Mapping and systematic appraisal of umbrella reviews in epidemiological research: a protocol for a meta-epidemiological study. Syst Rev.

[14] Pollock M (2023). Cochrane Handbook for Systematic Reviews of Interventions.

[15] Tricco AC, Straus SE, Ghaffar A, Langlois EV (2022). Rapid reviews for health policy and systems decision-making: more important than ever before. Syst Rev.

[16] Qureshi R, Shaughnessy D, Gill KAR, Robinson KA, Li T, Agai E (2023). Are ChatGPT and large language models “the answer” to bringing us closer to systematic review automation?. Syst Rev.

[17] Teperikidis L, Boulmpou A, Papadopoulos C, Biondi-Zoccai G (2024). Using ChatGPT to perform a systematic review: a tutorial. Minerva Cardiol Angiol.

[18] (2025). OpenAI.

[19] Elliott JH, Synnot A, Turner T (2017). Living systematic review: 1. Introduction-the why, what, when, and how. J Clin Epidemiol.

[20] Guyatt GH, Oxman AD, Vist GE (2008). GRADE: an emerging consensus on rating quality of evidence and strength of recommendations. BMJ.

[21] Conneely M, Leahy S, Dore L (2022). The effectiveness of interventions to reduce adverse outcomes among older adults following emergency department discharge: umbrella review. BMC Geriatr.

[22] Veronese N, Honvo G, Bruyère O (2023). Knee osteoarthritis and adverse health outcomes: an umbrella review of meta-analyses of observational studies. Aging Clin Exp Res.

[23] Shen Y, Liu D, Li S (2022). Effects of exercise on patients important outcomes in older people with sarcopenia: an umbrella review of meta-analyses of randomized controlled trials. Front Med (Lausanne).

[24] Musazadeh V, Kavyani Z, Mirhosseini N, Dehghan P, Vajdi M (2023). Effect of vitamin D supplementation on type 2 diabetes biomarkers: an umbrella of interventional meta-analyses. Diabetol Metab Syndr.

[25] Veronese N, Galvano D, D’Antiga F (2021). Interventions for reducing loneliness: an umbrella review of intervention studies. Health Soc Care Community.

[26] Veronese N, Smith L, Bolzetta F, Cester A, Demurtas J, Punzi L (2021). Efficacy of conservative treatments for hand osteoarthritis. Wien Klin Wochenschr.

[27] Marx W, Veronese N, Kelly JT (2021). The dietary inflammatory index and human health: an umbrella review of meta-analyses of observational studies. Adv Nutr.

[28] Gazzaniga G, Menichelli D, Scaglione F, Farcomeni A, Pani A, Pastori D (2023). Effect of digoxin on all-cause and cardiovascular mortality in patients with atrial fibrillation with and without heart failure: an umbrella review of systematic reviews and 12 meta-analyses. Eur J Clin Pharmacol.

[29] Higgins J (2023). Cochrane Handbook for Systematic Reviews of Interventions 2023.

[30] McKenzie JE, Brennan SE (2017). Overviews of systematic reviews: great promise, greater challenge. Syst Rev.

[31] Luchini C, Veronese N, Nottegar A (2021). Assessing the quality of studies in meta-research: review/guidelines on the most important quality assessment tools. Pharm Stat.

[32] Seehra J, Pandis N, Koletsi D, Fleming PS (2016). Use of quality assessment tools in systematic reviews was varied and inconsistent. J Clin Epidemiol.

[33] Bigna JJR, Um LN, Nansseu JRN (2016). A comparison of quality of abstracts of systematic reviews including meta-analysis of randomized controlled trials in high-impact general medicine journals before and after the publication of PRISMA extension for abstracts: a systematic review and meta-analysis. Syst Rev.

[34] Li T, Hua F, Dan S, Zhong Y, Levey C, Song Y (2020). Reporting quality of systematic review abstracts in operative dentistry: an assessment using the PRISMA for Abstracts guidelines. J Dent.

[35] El Ansari W, AlRumaihi K, El-Ansari K (2022). Reporting quality of abstracts of systematic reviews/meta-analyses: an appraisal of Arab Journal of Urology across 12 years: the PRISMA-Abstracts checklist. Arab J Urol.

[36] Zhong Y, Wang Y, Dan S (2023). The reporting quality of systematic review abstracts in leading general dental journals: a methodological study. J Evid Based Dent Pract.

[37] Aromataris E, Lockwood C, Porritt K, Pilla B JBI Manual for Evidence Synthesis. JBI Global Wiki.

[38] Brzezińska J (2015). The problem of zero cells in the analysis of contingency tables. KREM.

[39] Sapra RL, Saluja S (2021). Understanding statistical association and correlation. Current Medicine Research and Practice.

[40] Sullivan GM, Feinn R (2012). Using effect size-or why the P value is not enough. J Grad Med Educ.

[41] Demidenko E (2016). The P-value you can’t buy. Am Stat.

[42] Greenland S (2019). Valid P-values behave exactly as they should: some misleading criticisms of P-values and their resolution with S-values. Am Stat.

[43] Lu Y, Belitskaya-Levy I (2015). The debate about P-values. Shanghai Arch Psychiatry.

[44] Franco JVA, Arancibia M, Meza N, Madrid E, Kopitowski K (2020). Clinical practice guidelines: concepts, limitations and challenges. Medwave.

[45] Papatheodorou S (2019). Umbrella reviews: what they are and why we need them. Eur J Epidemiol.

[46] Shea BJ, Reeves BC, Wells G (2017). AMSTAR 2: a critical appraisal tool for systematic reviews that include randomised or non-randomised studies of healthcare interventions, or both. BMJ.

[47] Schlesinger S, Schwingshackl L, Neuenschwander M, Barbaresko J (2019). A critical reflection on the grading of the certainty of evidence in umbrella reviews. Eur J Epidemiol.

[48] Hultcrantz M, Rind D, Akl EA (2017). The GRADE Working Group clarifies the construct of certainty of evidence. J Clin Epidemiol.

[49] Borah R, Brown AW, Capers PL, Kaiser KA (2017). Analysis of the time and workers needed to conduct systematic reviews of medical interventions using data from the PROSPERO registry. BMJ Open.

[50] Hamel C, Michaud A, Thuku M (2021). Defining rapid reviews: a systematic scoping review and thematic analysis of definitions and defining characteristics of rapid reviews. J Clin Epidemiol.

[51] Clark J, Glasziou P, Del Mar C, Bannach-Brown A, Stehlik P, Scott AM (2020). A full systematic review was completed in 2 weeks using automation tools: a case study. J Clin Epidemiol.

[52] Tricco AC, EtienneV L, Straus SE (2017). Rapid Reviews to Strengthen Health Policy and Systems: A Practical Guide.

[53] Garritty C, Gartlehner G, Nussbaumer-Streit B (2021). Cochrane Rapid Reviews Methods Group offers evidence-informed guidance to conduct rapid reviews. J Clin Epidemiol.

[54] Garner P, Hopewell S, Chandler J (2016). When and how to update systematic reviews: consensus and checklist. BMJ.

[55] Hoffmeyer B, Fonnes S, Andresen K, Rosenberg J (2023). Use of inactive Cochrane reviews in academia: a citation analysis. Scientometrics.

[56] Beller EM, Chen JH, Wang UH, Glasziou PP (2013). Are systematic reviews up-to-date at the time of publication?. Syst Rev.

[57] Bashir R, Surian D, Dunn AG (2018). Time-to-update of systematic reviews relative to the availability of new evidence. Syst Rev.

[58] Heinen L, Goossen K, Lunny C, Hirt J, Puljak L, Pieper D (2024). The optimal approach for retrieving systematic reviews was achieved when searching MEDLINE and Epistemonikos in addition to reference checking: a methodological validation study. BMC Med Res Methodol.

[59] Bramer WM, Rethlefsen ML, Kleijnen J, Franco OH (2017). Optimal database combinations for literature searches in systematic reviews: a prospective exploratory study. Syst Rev.

[60] Aromataris E, Riitano D (2014). Systematic reviews: constructing a search strategy and searching for evidence. Am J Nurs.

[61] Yu X, Wu S, Sun Y (2024). Exploring the diverse definitions of “evidence”: a scoping review. BMJ Evid Based Med.

[62] Guimarães NS, Joviano-Santos JV, Reis MG, Chaves RRM, Observatory of Epidemiology, Nutrition, Health Research (OPENS) (2024). Development of search strategies for systematic reviews in health using ChatGPT: a critical analysis. J Transl Med.

[63] Ge L, Agrawal R, Singer M (2024). Leveraging artificial intelligence to enhance systematic reviews in health research: advanced tools and challenges. Syst Rev.

[64] Gusenbauer M, Haddaway NR (2020). Which academic search systems are suitable for systematic reviews or meta-analyses? Evaluating retrieval qualities of Google Scholar, PubMed, and 26 other resources. Res Synth Methods.

[65] Jia Y, Huang D, Wen J (2020). Assessment of language and indexing biases among Chinese-sponsored randomized clinical trials. JAMA Netw Open.

[66] Mao C, Li M (2020). Language bias among Chinese-sponsored randomized clinical trials in systematic reviews and meta-analyses-can anything be done?. JAMA Netw Open.

