# Supplementary material for: Validation of The Umbrella Collaboration for Tertiary Evidence Synthesis in Geriatrics: Mixed Methods Study
Source: JMIR Form Res. 2025 Jul 8;9:e75215. doi: 10.2196/75215 (PMC12262930; doi:10.2196/75215)
Supplement: Multimedia Appendix 1 [file formative-v9-e75215-s001.docx]

# Appendix 1. Practical Overview of The Umbrella Collaboration® Platform

The Umbrella Collaboration^®^ is an interactive, publicly accessible platform (<https://theumbrellacollaboration.org/>) that displays the results of tertiary evidence synthesis projects conducted using the TU^®^ methodology (Figure S1).

Figure S1:
Interface view: Homepage of The Umbrella Collaboration^®^


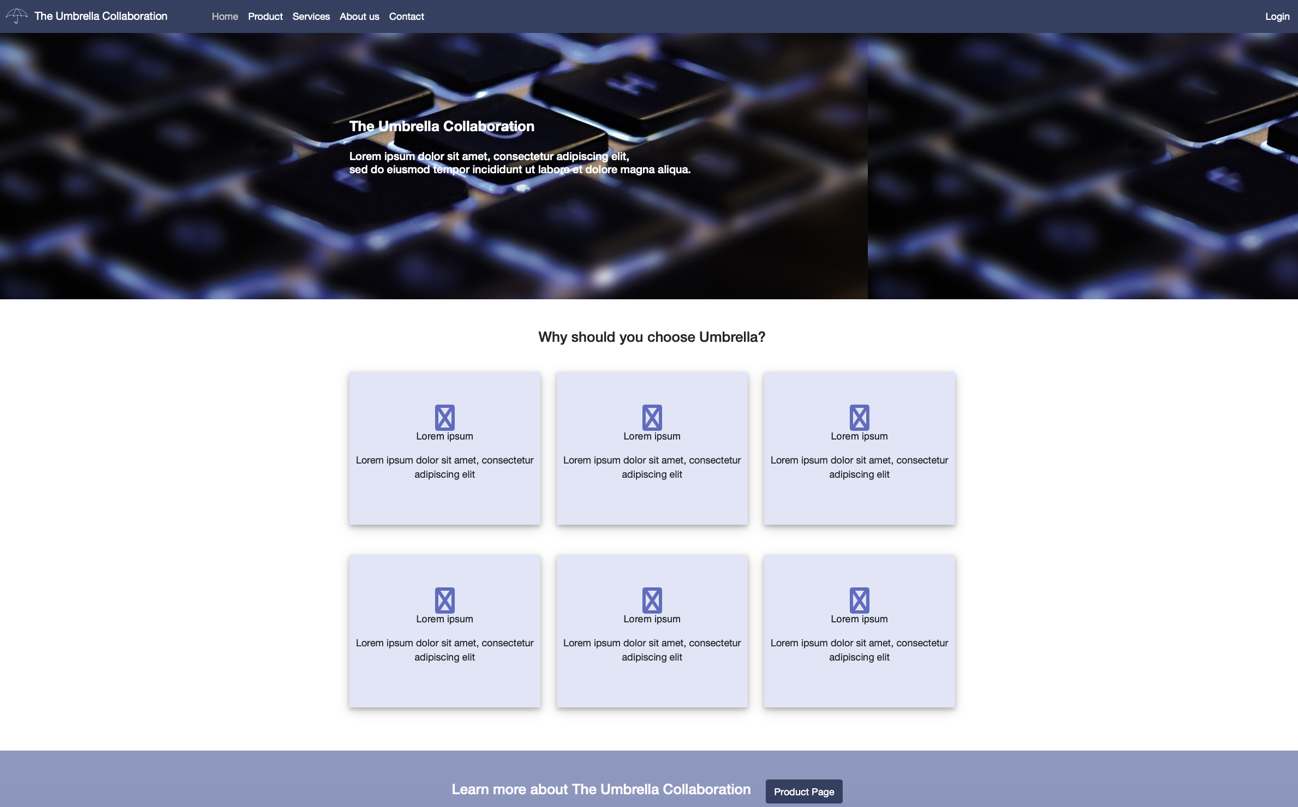


To access the results of TU^®^ projects, users must first register on the platform. Once registered, they can navigate to the “Medical Specialties” page (<https://theumbrellacollaboration.org/#/select-speciality>), which organizes all available projects according to a modified version of the European Union classification of medical specialties (Figure S2).

Figure S2
Interface view: List of specialties on The Umbrella Collaboration^®^ website


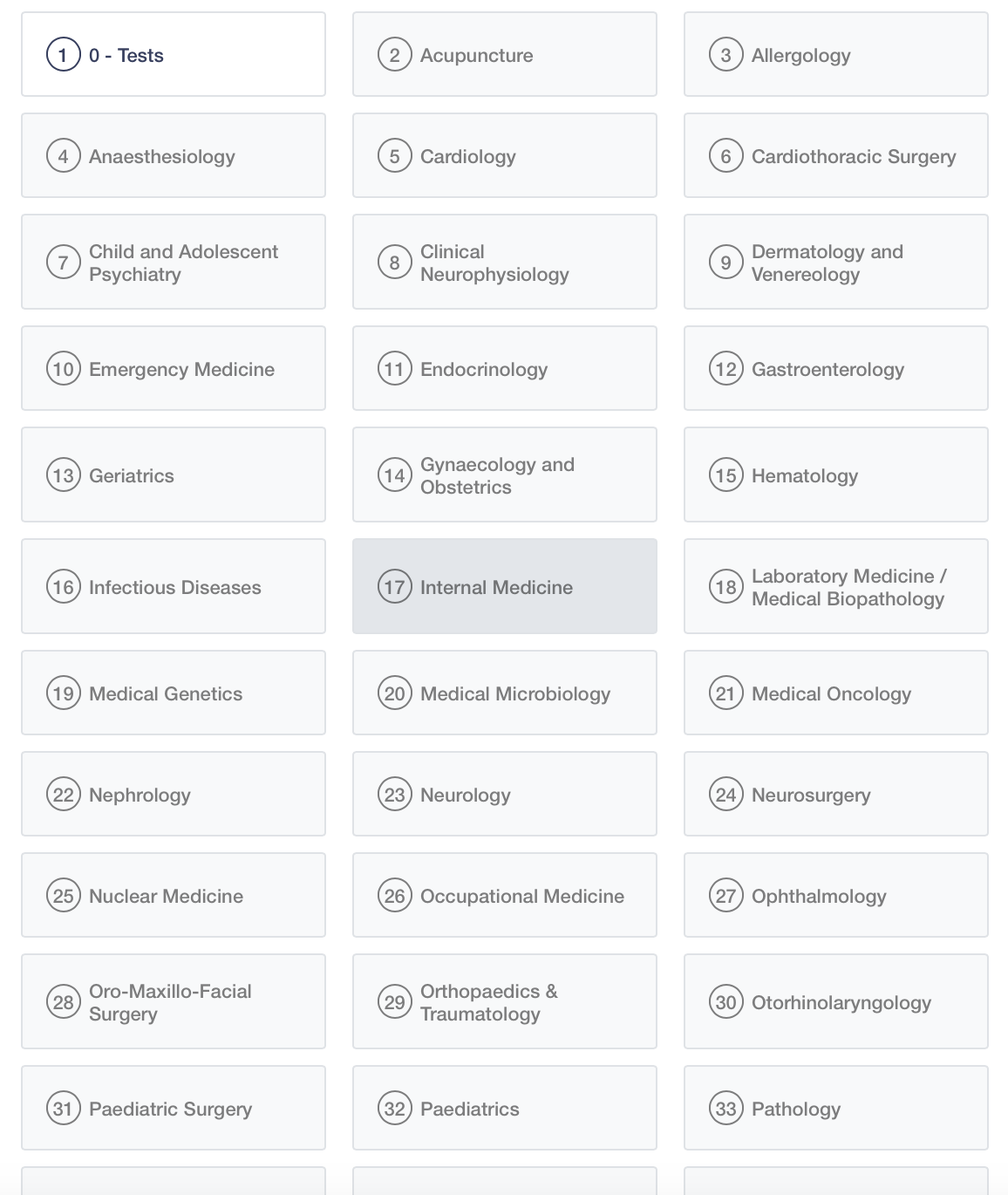


Within this list, the section labeled "Test" contains a collection of demonstration projects developed during the platform’s initial validation phase. Selecting this section reveals a list of projects, each displaying the results generated through TU^®^’s tertiary evidence synthesis process. Users can explore detailed data and findings by clicking on the title of any project (Figure S3).

Figure S3
Interface view: List of specialties on The Umbrella Collaboration^®^ website Project list under the "Test" section on The Umbrella Collaboration^®^


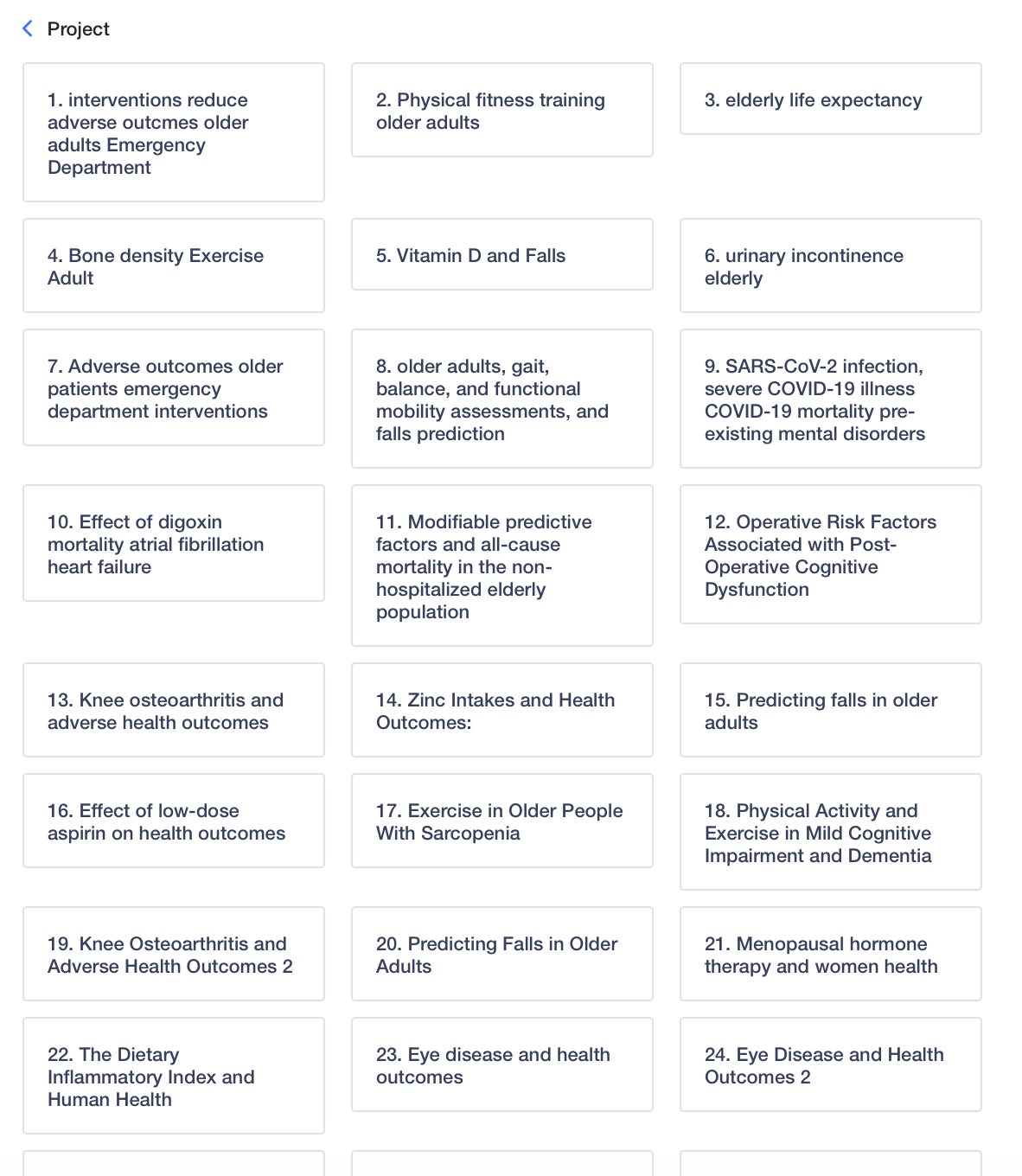


Projects can also be accessed directly via <https://theumbrellacollaboration.org/#/select-project>. There, users can view and select from the complete list of available projects. Upon clicking a project, the full results of the tertiary synthesis are displayed, enabling thorough exploration of each project’s evidence.

For example, selecting the project titled “Knee Osteoarthritis and Related Health Outcomes” brings users to the main results screen, labeled “Principal Bubble Plot” (Figure S4). This visualization consists of three main elements: (1) a summary list of all outcomes of interest (OoIs), (2) a legend explaining the meaning of icons and color codes, and (3) the bubble plot itself.

Shapes indicate statistical significance and directionality, triangles for significant results (oriented by effect direction) and circles for non-significant results. Colors represent effect size: red (trivial), orange (small), yellow (moderate), blue (large), and green (very large). The size of each figure reflects the number of primary studies included across the systematic reviews contributing to that OoI. The graph is structured into four quadrants indicating levels of certainty: Weak, Suggestive, Highly Suggestive, and Convincing. The y-axis indicates the number of systematic reviews contributing to each OoI.

Figure S4
Interface view: Principal Bubble Plot – Knee Osteoarthritis and Health Outcomes


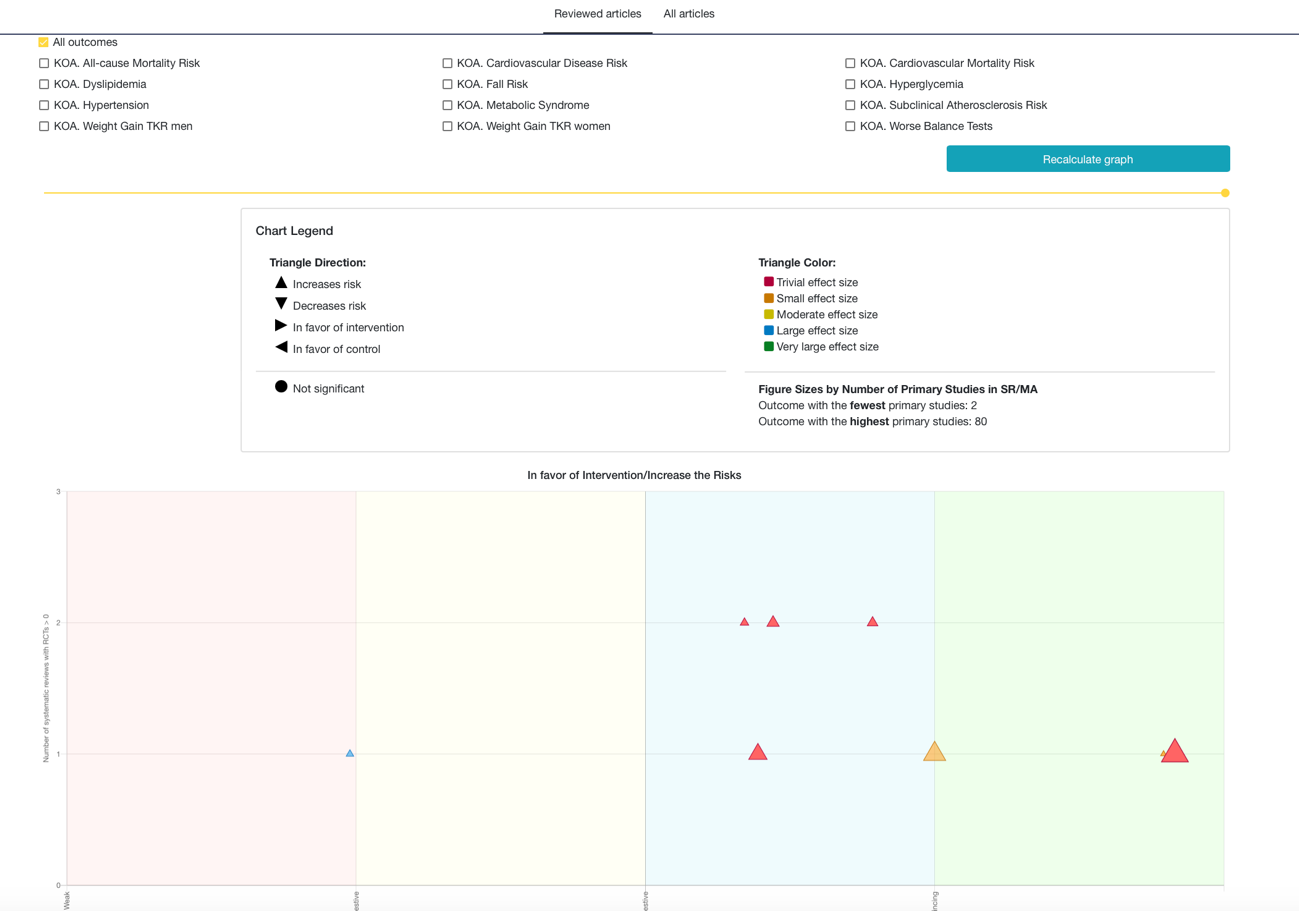


Hovering the cursor over any icon in the Principal Bubble Plot triggers a pop-up window with additional details about that specific OoI. This includes the OoI name (which acts as a hyperlink to deeper analyses), the number of systematic reviews and primary studies involved, the R_TU_ effect size and confidence intervals, and the certainty score based on sentiment analysis. This information helps users assess the strength and significance of each result (Figure S5).

Figure S5
Interface view: Pop-up display for the outcome “Subclinical Atherosclerosis Risk” in the Knee Osteoarthritis project


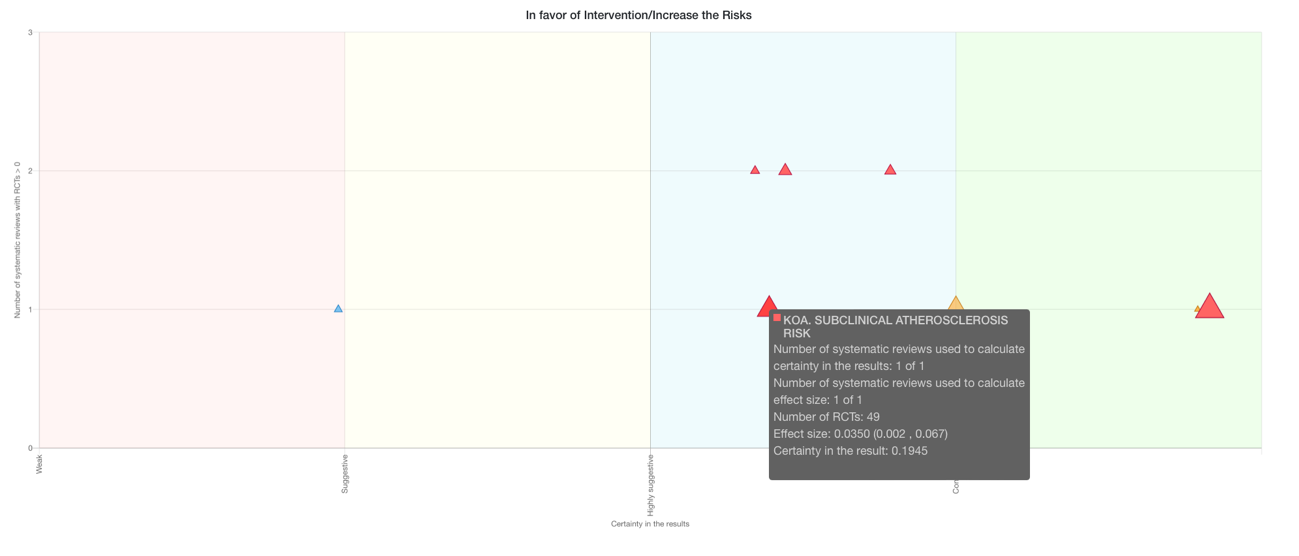


Clicking on a specific OoI opens three secondary visualizations: the Forest Plot, the Secondary Bubble Plot, and the Systematic Review List.

The Forest Plot presents the R_TU_ effect sizes from each SR/MA contributing to that OoI, in a format similar to traditional meta-analyses. The horizontal axis spans from -1 to +1, with 0 as the null value. Confidence intervals that cross zero indicate non-significance. Each figure (triangle or circle) is colored according to effect size and linked to the abstract in PubMed. At the bottom of the graph, TU^®^ displays the aggregated R_TU_ value calculated from all included SRs/MAs (Figure S6).

Figure S6
Interface view: Forest Plot for “Subclinical Atherosclerosis Risk” in the Knee Osteoarthritis project


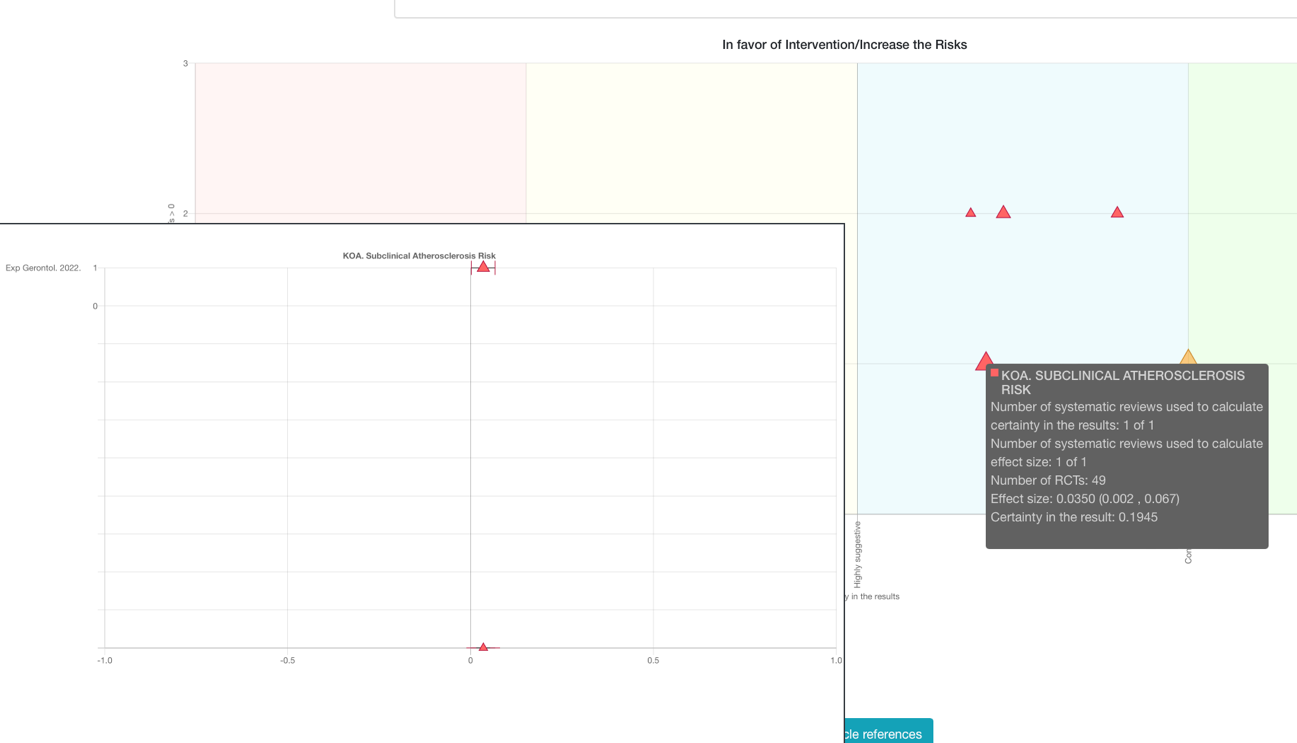


The Secondary Bubble Plot displays each contributing individual OoI from the SRs/MAs included in the synthesis. The x-axis represents certainty (sentiment score from -1 to +1), and the y-axis the number of primary studies. Shapes, orientation, color, and size follow the same visual coding as the main bubble plot (Figure S7).

Figure S7
Interface view: Secondary Bubble Plot and list of SRs/MAs for “Subclinical Atherosclerosis Risk” in the Knee Osteoarthritis project


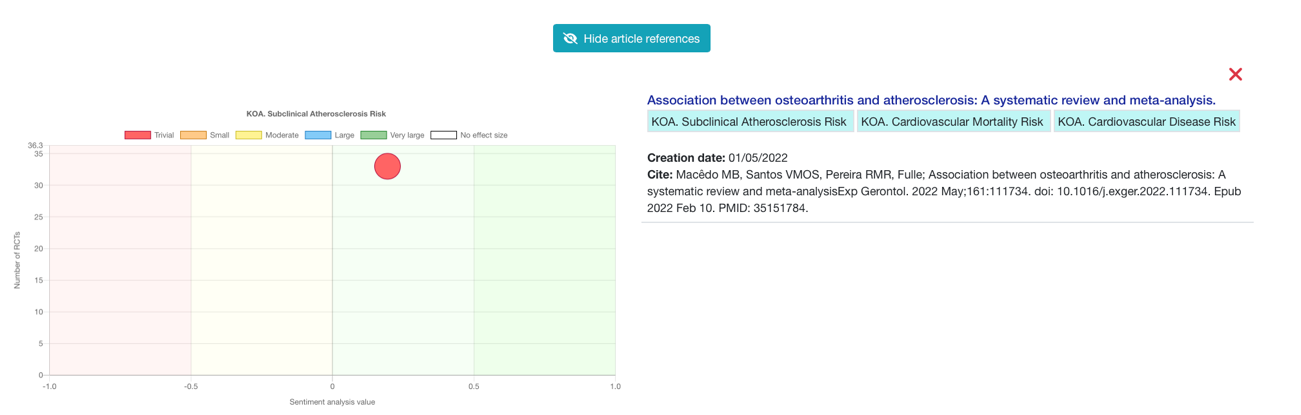


Finally, the Systematic Review List provides key metadata for each SR/MA included in the selected OoI. This includes title (linked to the PubMed abstract), list of outcomes evaluated in the review, and full citation with publication date. This interactive format supports an in-depth, user-friendly exploration of each synthesized result.
