# Supplementary material for: Validation of The Umbrella Collaboration for Tertiary Evidence Synthesis in Geriatrics: Mixed Methods Study
Source: JMIR Form Res. 2025 Jul 8;9:e75215. doi: 10.2196/75215 (PMC12262930; doi:10.2196/75215)
Supplement: Multimedia Appendix 2 [file formative-v9-e75215-s002.docx]

Appendix 2

Table S2
Umbrella reviews that met the inclusion criteria but were not selected for comparative analysis

| Initial TUR search  36/111 | TURs included for comparison  8/36  (nº project) | Author (año) | Title TUR |
| --- | --- | --- | --- |
| 1 |  | Visser, 2022  (1) | Effectiveness and characteristics of physical fitness training on aerobic fitness in vulnerable older adults: an umbrella review of systematic reviews. |
| 2 |  | Yuan, 2022  (2) | Modifiable predictive factors and all-cause mortality in the non-hospitalized elderly population: An umbrella review of meta-analyses. |
| 3 |  | Travica, 2023  (3) | Peri-Operative Risk Factors Associated with Post-Operative Cognitive Dysfunction (POCD): An Umbrella Review of Meta-Analyses of Observational Studies. |
| 4 |  | Li, 2022  (4) | Zinc Intakes and Health Outcomes: An Umbrella Review. |
| 5 |  | Beck Jepsen, 2022  (5) | Predicting falls in older adults: an umbrella review of instruments assessing gait, balance, and functional mobility. |
| 6 |  | Veronese, 2020  (6) | Effect of low-dose aspirin on health outcomes: An umbrella review of systematic reviews and meta-analyses. |
| 7 |  | Soysal, 2023  (7) | The impact of urinary incontinence on multiple health outcomes: an umbrella review of meta-analysis of observational studies. |
| 8 |  | Zhang, 2021  (8) | Menopausal hormone therapy and women's health: An umbrella review. |
| 9 |  | Trott, 2022  (9) | Eye disease and mortality, cognition, disease, and modifiable risk factors: an umbrella review of meta-analyses of observational studies. |
| 10 |  | Celotto, 2021  (10) | An umbrella review of systematic reviews with meta-analyses evaluating positive and negative outcomes of Hydroxychloroquine and chloroquine therapy. |
| 11 |  | Demurtas, 2021  (11) | Stem cells for treatment of cardiovascular diseases: An umbrella review of randomized controlled trials. |
| 12 |  | Demurtas, 2020  (12) | Physical Activity and Exercise in Mild Cognitive Impairment and Dementia: An Umbrella Review of Intervention and Observational Studies. |
| 13 |  | Obaid, 2020  (13) | Can we prevent poststroke cognitive impairment? An umbrella review of risk factors and treatments. |
| 14 |  | Veronese, 2020  (14) | Glucosamine sulphate: an umbrella review of health outcomes. |
| 15 |  | Trott, 2021  (15) | Hearing impairment and diverse health outcomes: An umbrella review of meta-analyses of observational studies. |
| 16 |  | Neil-Sztramko, 2022  (16) | Community-based group physical activity and/or nutrition interventions to promote mobility in older adults: an umbrella review. |
| 17 |  | Antonazzo, 2022  (17) | Impact of arterial hypertension and its management strategies on cognitive function and dementia: a comprehensive umbrella review. |
| 18 |  | Demurtas, 2020  (18) | The efficacy and safety of influenza vaccination in older people: An umbrella review of evidence from meta-analyses of both observational and randomized controlled studies. |
| 19 |  | Conroy, 2020  (19) | In-hospital interventions for reducing readmissions to acute care for adults aged 65 and over: An umbrella review. |
| 20 |  | Gielen, 2021  (20) | Nutritional interventions to improve muscle mass, muscle strength, and physical performance in older people: an umbrella review of systematic reviews and meta-analyses. |
| 21 |  | Biondi-Zoccai, 2020  (21) | Oral antiplatelet therapy in the elderly undergoing percutaneous coronary intervention: an umbrella review. |
| 22 |  | Ofori-Asenso, 2020  (22) | Frailty Confers High Mortality Risk across Different Populations: Evidence from an Overview of Systematic Reviews and Meta-Analyses. |
| 23 |  | Veronese, 2019  (23) | Sarcopenia and health-related outcomes: an umbrella review of observational studies. |
| 24 |  | Beckwée, 2019  (24) | Exercise Interventions for the Prevention and Treatment of Sarcopenia. A Systematic Umbrella Review. |
| 25 |  | De Spiegeleer, 2018  (25) | Pharmacological Interventions to Improve Muscle Mass, Muscle Strength and Physical Performance in Older People: An Umbrella Review of Systematic Reviews and Meta-analyses. |
| 26 |  | Jadczak, 2018  (26) | Effectiveness of exercise interventions on physical function in community-dwelling frail older people: an umbrella review of systematic reviews. |
| 27 |  | Poscia, 2018  (27) | Effectiveness of nutritional interventions addressed to elderly persons: umbrella systematic review with meta-analysis. |
| 28 |  | Stubbs, 2015  (28) | What works to prevent falls in older adults dwelling in long term care facilities and hospitals? An umbrella review of meta-analyses of randomised controlled trials. |

1. Visser D, Wattel EM, Gerrits KHL, Wouden JC van der, Meiland FJM, Groot AJ de, et al. Effectiveness and characteristics of physical fitness training on aerobic fitness in vulnerable older adults: an umbrella review of systematic reviews. BMJ Open [Internet]. 2022 May 1 [cited 2024 Nov 6];12(5):e058056. Available from: https://bmjopen.bmj.com/content/12/5/e058056

2. Yuan Y, Lin S, Lin W, Huang F, Zhu P. Modifiable predictive factors and all-cause mortality in the non-hospitalized elderly population: An umbrella review of meta-analyses. Exp Gerontol [Internet]. 2022 Jun 15 [cited 2024 Nov 6];163:111792. Available from: https://www.sciencedirect.com/science/article/pii/S0531556522001000

3. Travica N, Lotfaliany M, Marriott A, Safavynia SA, Lane MM, Gray L, et al. Peri-Operative Risk Factors Associated with Post-Operative Cognitive Dysfunction (POCD): An Umbrella Review of Meta-Analyses of Observational Studies. J Clin Med [Internet]. 2023 Jan [cited 2024 Nov 6];12(4):1610. Available from: https://www.mdpi.com/2077-0383/12/4/1610

4. Li J, Cao D, Huang Y, Chen B, Chen Z, Wang R, et al. Zinc Intakes and Health Outcomes: An Umbrella Review. Front Nutr [Internet]. 2022 Feb 8 [cited 2024 Nov 6];9. Available from: https://www.frontiersin.org/journals/nutrition/articles/10.3389/fnut.2022.798078/full

5. Beck Jepsen D, Robinson K, Ogliari G, Montero-Odasso M, Kamkar N, Ryg J, et al. Predicting falls in older adults: an umbrella review of instruments assessing gait, balance, and functional mobility. BMC Geriatr [Internet]. 2022 Jul 25 [cited 2024 Nov 6];22(1):615. Available from: https://doi.org/10.1186/s12877-022-03271-5

6. Veronese N, Demurtas J, Thompson T, Solmi M, Pesolillo G, Celotto S, et al. Effect of low-dose aspirin on health outcomes: An umbrella review of systematic reviews and meta-analyses. Br J Clin Pharmacol [Internet]. 2020 [cited 2024 Nov 6];86(8):1465–75. Available from: https://onlinelibrary.wiley.com/doi/abs/10.1111/bcp.14310

7. Soysal P, Veronese N, Ippoliti S, Pizzol D, Carrie AM, Stefanescu S, et al. The impact of urinary incontinence on multiple health outcomes:: an umbrella review of meta-analysis of observational studies. Aging Clin Exp Res [Internet]. 2023 Jan 13 [cited 2024 Nov 24];35(3):479–95. Available from: http://www.scopus.com/inward/record.url?scp=85146238196&partnerID=8YFLogxK

8. Zhang GQ, Chen JL, Luo Y, Mathur MB, Anagnostis P, Nurmatov U, et al. Menopausal hormone therapy and women’s health: An umbrella review. PLOS Med [Internet]. 2021 Aug 2 [cited 2024 Nov 6];18(8):e1003731. Available from: https://journals.plos.org/plosmedicine/article?id=10.1371/journal.pmed.1003731

9. Trott M, Smith L, Veronese N, Pizzol D, Barnett Y, Gorely T, et al. Eye disease and mortality, cognition, disease, and modifiable risk factors: an umbrella review of meta-analyses of observational studies. Eye [Internet]. 2022 Feb [cited 2024 Nov 6];36(2):369–78. Available from: https://www.nature.com/articles/s41433-021-01684-x

10. Celotto S, Veronese N, Barbagallo M, Ometto F, Smith L, Pardhan S, et al. An umbrella review of systematic reviews with meta-analyses evaluating positive and negative outcomes of Hydroxychloroquine and chloroquine therapy. Int J Infect Dis [Internet]. 2021 Feb 1 [cited 2024 Nov 6];103:599–606. Available from: https://www.ijidonline.com/article/S1201-9712(20)32541-8/fulltext

11. Demurtas J, Fanelli GN, Romano SL, Solari M, Yang L, Soysal P, et al. Stem cells for treatment of cardiovascular diseases: An umbrella review of randomized controlled trials. Ageing Res Rev [Internet]. 2021 May 1 [cited 2024 Nov 24];67:101257. Available from: https://www.sciencedirect.com/science/article/pii/S1568163721000040

12. Demurtas J, Schoene D, Torbahn G, Marengoni A, Grande G, Zou L, et al. Physical Activity and Exercise in Mild Cognitive Impairment and Dementia: An Umbrella Review of Intervention and Observational Studies. J Am Med Dir Assoc [Internet]. 2020 Oct 1 [cited 2024 Nov 24];21(10):1415-1422.e6. Available from: https://www.sciencedirect.com/science/article/pii/S1525861020307374

13. Obaid M, Douiri A, Flach C, Prasad V, Marshall I. Can we prevent poststroke cognitive impairment? An umbrella review of risk factors and treatments. BMJ Open [Internet]. 2020 Sep 1 [cited 2024 Nov 6];10(9):e037982. Available from: https://bmjopen.bmj.com/content/10/9/e037982

14. Veronese N, Demurtas J, Smith L, Reginster JY, Bruyère O, Beaudart C, et al. Glucosamine sulphate: an umbrella review of health outcomes. Ther Adv Musculoskelet Dis [Internet]. 2020 Jan 1 [cited 2024 Nov 6];12:1759720X20975927. Available from: https://doi.org/10.1177/1759720X20975927

15. Trott M, Smith L, Xiao T, Veronese N, Koyanagi A, Jacob L, et al. Hearing impairment and diverse health outcomes. Wien Klin Wochenschr [Internet]. 2021 Oct 1 [cited 2024 Nov 6];133(19):1028–41. Available from: https://doi.org/10.1007/s00508-021-01893-0

16. Neil-Sztramko SE, Teggart K, Moore C, Sherifali D, Fitzpatrick-Lewis D, Coletta G, et al. Community-based group physical activity and/or nutrition interventions to promote mobility in older adults: an umbrella review. BMC Geriatr [Internet]. 2022 Jun 29 [cited 2024 Nov 6];22(1):539. Available from: https://doi.org/10.1186/s12877-022-03170-9

17. Antonazzo B, Marano G, Romagnoli E, Ronzoni S, Frati G, Sani G, et al. Impact of arterial hypertension and its management strategies on cognitive function and dementia: a comprehensive umbrella review. Minerva Cardiol Angiol. 2022 Jun;70(3):285–97.

18. Demurtas J, Celotto S, Beaudart C, Sanchez-Rodriguez D, Balci C, Soysal P, et al. The efficacy and safety of influenza vaccination in older people: An umbrella review of evidence from meta-analyses of both observational and randomized controlled studies. Ageing Res Rev [Internet]. 2020 Sep 1 [cited 2024 Nov 6];62:101118. Available from: https://www.sciencedirect.com/science/article/pii/S1568163720302531

19. Conroy T, Heuzenroeder L, Feo R. In-hospital interventions for reducing readmissions to acute care for adults aged 65 and over: An umbrella review. Int J Qual Health Care [Internet]. 2020 Aug 1 [cited 2024 Nov 6];32(7):414–30. Available from: https://doi.org/10.1093/intqhc/mzaa064

20. Gielen E, Beckwée D, Delaere A, De Breucker S, Vandewoude M, Bautmans I, et al. Nutritional interventions to improve muscle mass, muscle strength, and physical performance in older people: an umbrella review of systematic reviews and meta-analyses. Nutr Rev [Internet]. 2021 Jan 9 [cited 2024 Nov 6];79(2):121–47. Available from: https://doi.org/10.1093/nutrit/nuaa011

21. Biondi-Zoccai G, Antonazzo B, Giordano A, Versaci F, Frati G, Ronzoni S, et al. Oral antiplatelet therapy in the elderly undergoing percutaneous coronary intervention: an umbrella review. J Thorac Dis [Internet]. 2020 Apr [cited 2024 Nov 6];12(4). Available from: https://jtd.amegroups.org/article/view/35967

22. Ofori-Asenso R, Chin KL, Sahle BW, Mazidi M, Zullo AR, Liew D. Frailty Confers High Mortality Risk across Different Populations: Evidence from an Overview of Systematic Reviews and Meta-Analyses. Geriatrics [Internet]. 2020 Mar [cited 2024 Nov 6];5(1):17. Available from: https://www.mdpi.com/2308-3417/5/1/17

23. Veronese N, Demurtas J, Soysal P, Smith L, Torbahn G, Schoene D, et al. Sarcopenia and health-related outcomes: an umbrella review of observational studies. Eur Geriatr Med [Internet]. 2019 Dec 1 [cited 2024 Nov 6];10(6):853–62. Available from: https://doi.org/10.1007/s41999-019-00233-w

24. Beckwée D, Delaere A, Aelbrecht S, Baert V, Beaudart C, Bruyere O, et al. Exercise Interventions for the Prevention and Treatment of Sarcopenia. A Systematic Umbrella Review. J Nutr Health Aging [Internet]. 2019 Jun 1 [cited 2024 Nov 6];23(6):494–502. Available from: https://www.sciencedirect.com/science/article/pii/S1279770723011958

25. De Spiegeleer A, Beckwée D, Bautmans I, Petrovic M, Bautmans I, Beaudart C, et al. Pharmacological Interventions to Improve Muscle Mass, Muscle Strength and Physical Performance in Older People: An Umbrella Review of Systematic Reviews and Meta-analyses. Drugs Aging [Internet]. 2018 Aug 1 [cited 2024 Nov 6];35(8):719–34. Available from: https://doi.org/10.1007/s40266-018-0566-y

26. Jadczak AD, Makwana N, Luscombe-Marsh N, Visvanathan R, Schultz TJ. Effectiveness of exercise interventions on physical function in community-dwelling frail older people: an umbrella review of systematic reviews. JBI Evid Synth [Internet]. 2018 Mar [cited 2024 Nov 6];16(3):752. Available from: https://journals.lww.com/jbisrir/abstract/2018/03000/effectiveness_of_exercise_interventions_on.17.aspx

27. Poscia A, Milovanovic S, La Milia DI, Duplaga M, Grysztar M, Landi F, et al. Effectiveness of nutritional interventions addressed to elderly persons: umbrella systematic review with meta-analysis. Eur J Public Health [Internet]. 2018 Apr 1 [cited 2024 Nov 6];28(2):275–83. Available from: https://doi.org/10.1093/eurpub/ckx199

28. Stubbs B, Denkinger MD, Brefka S, Dallmeier D. What works to prevent falls in older adults dwelling in long term care facilities and hospitals? An umbrella review of meta-analyses of randomised controlled trials. Maturitas. 2015 Jul;81(3):335–42.
